# Ultraviolet Fluorescence Spectra of Fingerprints

**DOI:** 10.1100/tsw.2005.43

**Published:** 2005-05-02

**Authors:** Naoki Saitoh, Norimitsu Akiba

**Affiliations:** Second Forensic Science Division, National Research Institute of Police Science, 6-3-1 Kashiwanoha, Kashiwa, Chiba 277-0882, Japan

**Keywords:** fingerprint, ultraviolet fluorescence, Nd-YAG laser, time-resolved spectroscopy

## Abstract

We have studied inherent fluorescence spectra and imaging of fingerprints in the deep ultraviolet (UV) region with a nanosecond-pulsed Nd-YAG laser system that consists of a tunable laser, a cooled CCD camera, and a grating spectrometer. In this paper, we have studied UV fluorescence spectra of fingerprints under 266-nm illumination. Fluorescence spectra of fingerprints have two main peaks, around 330 nm (peak A) and 440 nm (peak B). At first, when a fingerprint has just been pressed, peak A is dominant. However, its intensity reduces as the total illumination time increases. On the other hand, peak B is weak at first. It appears after enough 266-nm illumination and its intensity increases as time elapses. After 3 h of illumination, peak A almost diminishes and peak B becomes dominant. By leaving the fingerprint under a fluorescent lamp in a room without laser illumination, peak A can be restored partly, while the intensity of peak B still increases.Time-resolved fluorescence spectra were also measured for these two peaks. The lifetime of each peak is 2.0 nsec (peak A) and 6.2 nsec (peak B) on average. Both peaks seem to consist of several components with different lifetimes. In the case of peak A, the 330-nm peak decays fast and a new component at 360 nm becomes dominant when the delay time exceeds 20 nsec. In the case of peak B, unlike peak A, no clear peak separation is observed, but the peak position seems to move from 440 to 460 nm when the delay time becomes larger.

## INTRODUCTION

Detection of fingerprints using inherent fluorescence with laser was begun in 1976 by Dalrymple et al.[1]. Since then, many reports on laser detection of fingerprints have been published[2,3,4]. Most of them dealt with visible fluorescence and preprocessing procedures with various chemicals to enhance sensibility since the fluorescence intensity of fingerprints is weak and substrate materials emit strong fluorescence. Menzel[2] referred to the deep ultraviolet (UV) fluorescence, but wrote that visible fluorescence is more appropriate for detecting latent fingerprints since standard light sources cannot effectively excite deep UV fluorescence. In order to obtain UV fluorescence of fingerprints, it is necessary to excite with a strong light source, for example, a laser. Bramble et al.[5] showed that a 266-nm illumination of Nd-YAG laser is effective to visualize fingerprints on white papers. In addition, Bramble[6] measured three-dimensional

UV fluorescence spectra in which excitation wavelength, emission wavelength, and the fluorescence intensity are displayed together and showed that the fluorescence spectra of fingerprints ranges from 310–380 nm. He also showed that the excitation range for fingerprints is 260–300 nm. In this paper, we have studied UV fluorescence spectra of fingerprints excited with 266-nm illumination in detail with our new imaging system. In a future study, imaging fingerprints in the UV will be reported.

## EXPERIMENT

The block diagram of our system is depicted in Fig. 1. The system consists of a pulsed Nd-YAG laser (Continuum, Powerlite Precision 8010), OPO (Optical Parametric Oscillator) + Doubler (Continuum Panther EXST-1), a cooled CCD camera with an image intensifier (ICCD camera)(Roper Scientific, PI-MAX 1K-RB-FG-43), and a spectrometer (Acton Research Corporation, SpectraPro-300i). This laser is a tunable laser. The third harmonic wave of the Nd-YAG laser, whose wavelength is 355 nm, is input to OPO with a BBO ($BaB_2O_4$) crystal that generates light with broad wavelength from 410–2550 nm. Then the frequency is doubled with a Doubler unit. The resultant wavelength ranges from 215–420 nm. In this experiment, we used a 266-nm wavelength, which is equivalent to the fourth harmonic wave of the Nd-YAG laser, to excite the fluorescence of samples. The frequency of the laser pulse is 10 Hz and the width of the individual pulse and its energy are about 5 nsec and 4 mJ, respectively. Laser pulses were irradiated onto samples after expanding the beam with a beam expander or a holographic diffuser. With this system, time-resolved fluorescence spectra can be measured using the fast-gated image intensifier. The image intensifier can be gated faster than 2 nsec. The gating control of exposure time of the ICCD camera or delay time of observation is done by a PTG (programming timing generator) and a personal computer.

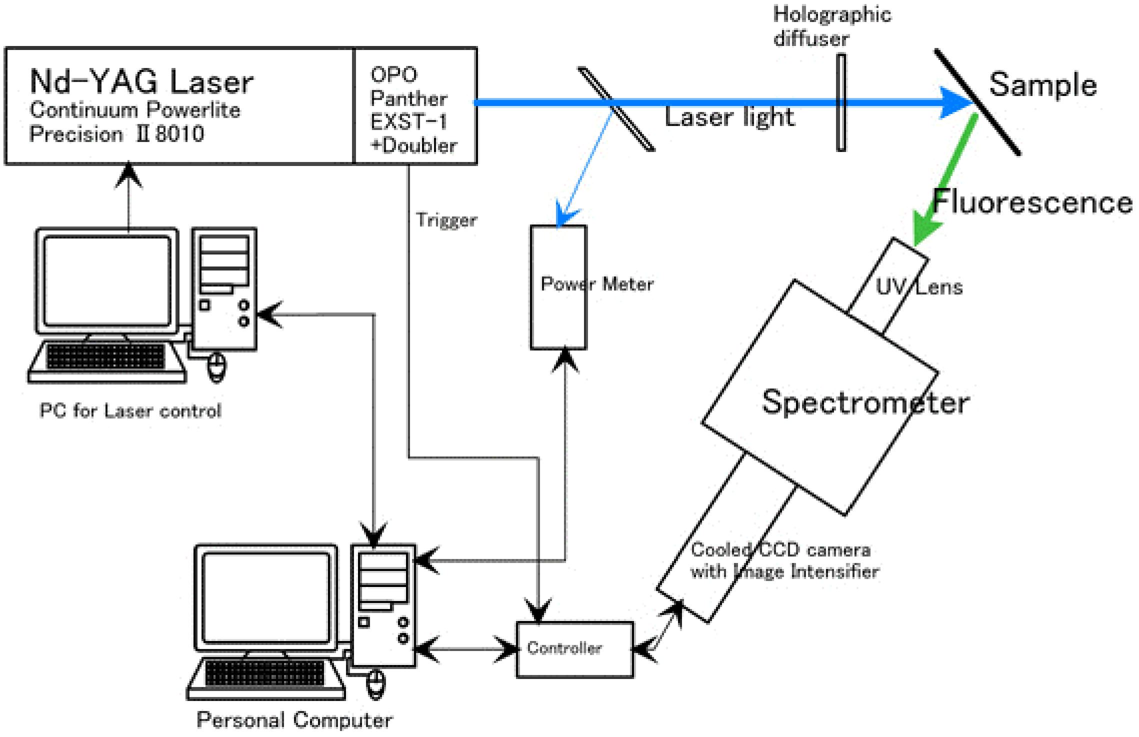

**FIGURE 1.** Block diagram of the time-resolving fluorescence imaging and spectroscopy system.

A fingerprint was pressed on a quartz glass that does not emit fluorescence. Before pressing the fingerprint, fingers were washed with soap and dried in the air. The fingerprint was pressed after wiping forehead or nose. Fluorescence was collected with a UV lens and put into the spectrometer that has a grating monochrometer with 1200 g/mm.

Spectra are measured by the ICCD camera attached to the spectrometer with the gating control. This CCD has 1024 × 1024 pixels of 13- × 13-μm pixel size. The wavelength resolution is 0.137056 nm per pixel. Therefore, for example, wavelength range is from 279.242–420.438 nm when the center of monochrometer is set to 350 nm. Obtained spectra were digitized in 16 bit. In order to suppress noise, the CCD is cooled to –20ºC. In addition, background spectra that consist of noise can be subtracted at each measurement. In case of measurement of fingerprint fluorescence, it is necessary to accumulate many measurements since fluorescence intensity is weak.

## FLUORESCENCE SPECTRA OF FINGERPRINT

In Fig. 2, a typical UV fluorescence spectrum of a fingerprint is shown. This spectrum was obtained with a 10-nsec delay from the laser pulse excitation after 20-min illumination. As is seen from Fig. 2, the spectra have two main peaks, one is located at around 330 nm and the other is at around 440 nm. In this paper, we call the peak around 330 nm peak A and the peak around 440 nm peak B. Peak A ranges from 270–400 nm. Peak B ranges from 380 to over 500 nm. However, this spectrum varies with 266-nm illumination. Bramble et al.[5] wrote that fluorescence at around 350 nm reduced with 266-nm illumination. He used the spectral component at a 350-nm wavelength using a band-pass filter of 350 nm to visualize the fluorescence image on a white paper and found that the fluorescence intensity became weak after continuous illumination. So we examined the time characteristics of the spectrum next.

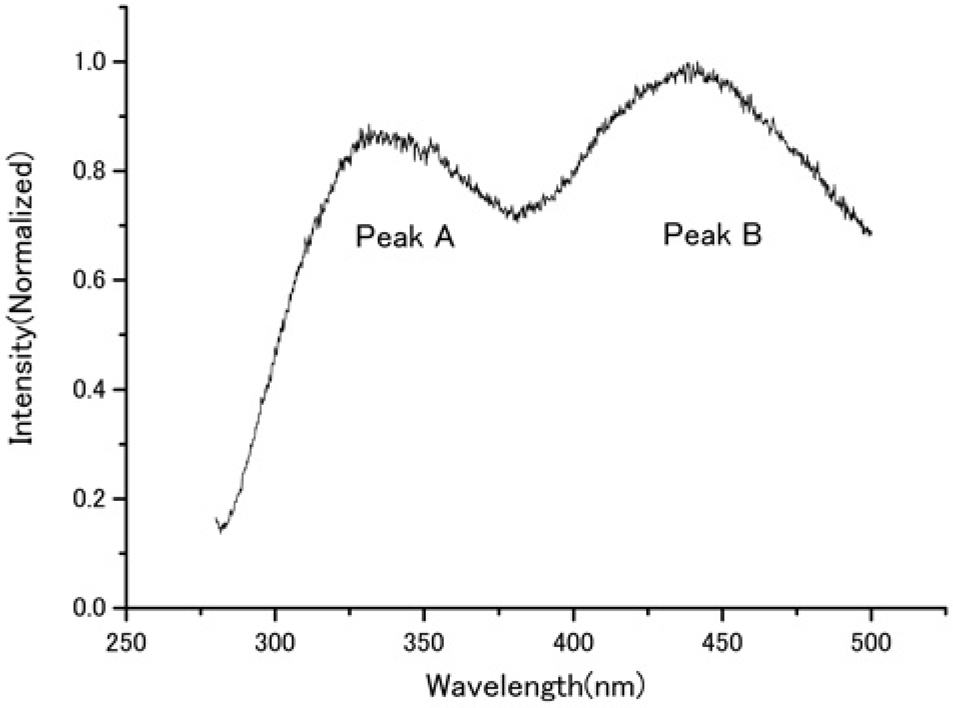

**FIGURE 2**. Typical UV fluorescence spectrum of a fingerprint on quartz glass excited by 266-nm laser light.

## TIME VARIATION OF FLUORESCENCE UNDER 266-NM ILLUMINATION

In Fig. 3, fluorescence spectra were shown for various illumination durations. The wavelength range is from 330–470 nm. The wavelength range of Fig. 3 is narrower than that of Fig. 2. In Fig. 2, two spectra were glued to achieve broad wavelength range in one spectrum. However, it is difficult to glue two spectra for every measurement because fluorescence spectra are varied by 266-nm illumination. Therefore in Fig. 3, the total spectral profile is not shown for both peak A and peak B. The total profile of peak A and peak B is shown in Figs. 4 and 6, respectively. As is seen from Fig. 3, when the fingerprint is new or has just been pressed, peak A dominates fluorescence. As the illumination time increases, the intensity of peak A reduces and after about 90 min, peak A almost seems to be eliminated. On the other hand, peak B seems to be weak at first, but then increases its intensity slightly with 266-nm illumination.

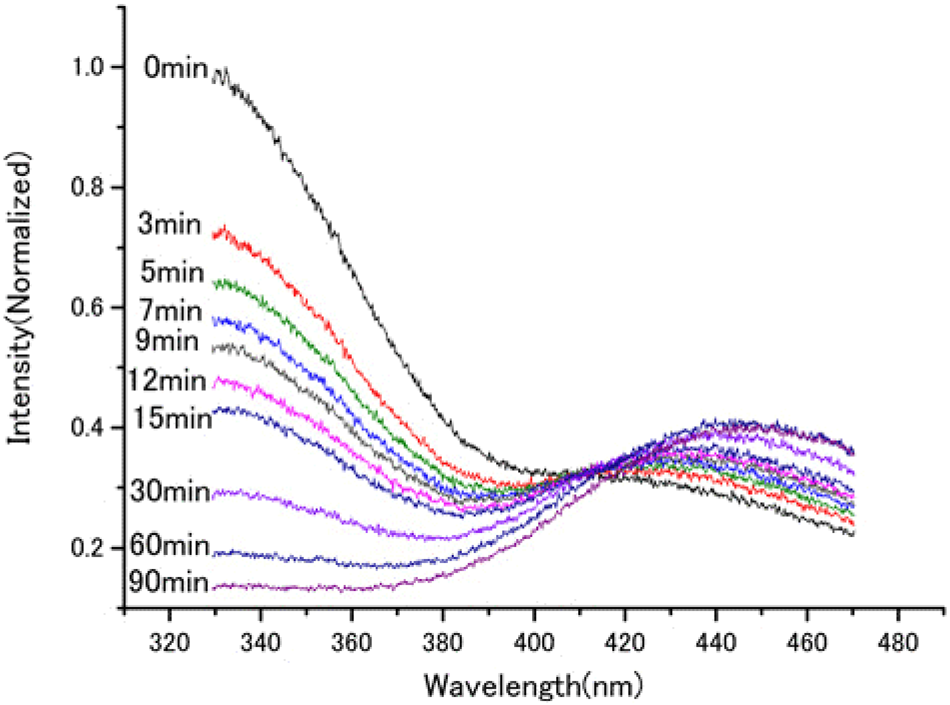

**FIGURE 3.** Time variation of fluorescence spectra of fingerprints. These spectra were measured with a 10-nsec delay time.

Peak A diminishes after 90-min illumination, but this does not mean that fluorescence corresponding to peak A completely disappears. In Fig. 4, the details of the fluorescence spectra of peak A are shown for various illumination durations. This peak shows its maximum at about 330 nm, but it seems to consist of several components. As the illumination time increases, the main peak moves to a lower position. In Fig. 5, the spectrum after 210-min illumination is shown. Eventually after 3 h, the peak position becomes about 305 nm, which means that the component at 330 nm reduces rapidly while the component at 305 nm remains.

In Fig. 6, the details of peak B are shown for various illumination durations from just pressed to 150 min. The intensity of peak B seems to increase as the illumination time increases. After 150-min illumination, illumination was stopped and the sample was left in a room under a fluorescent lamp for 3 days. Peak intensity doubles with this process.

In the case of peak A, its reduced intensity with 266-nm illumination was partly restored (Fig. 7) after 4 days during which no illumination was given. As can be seen in Fig. 7, several spectral components that consist of peak A appeared.

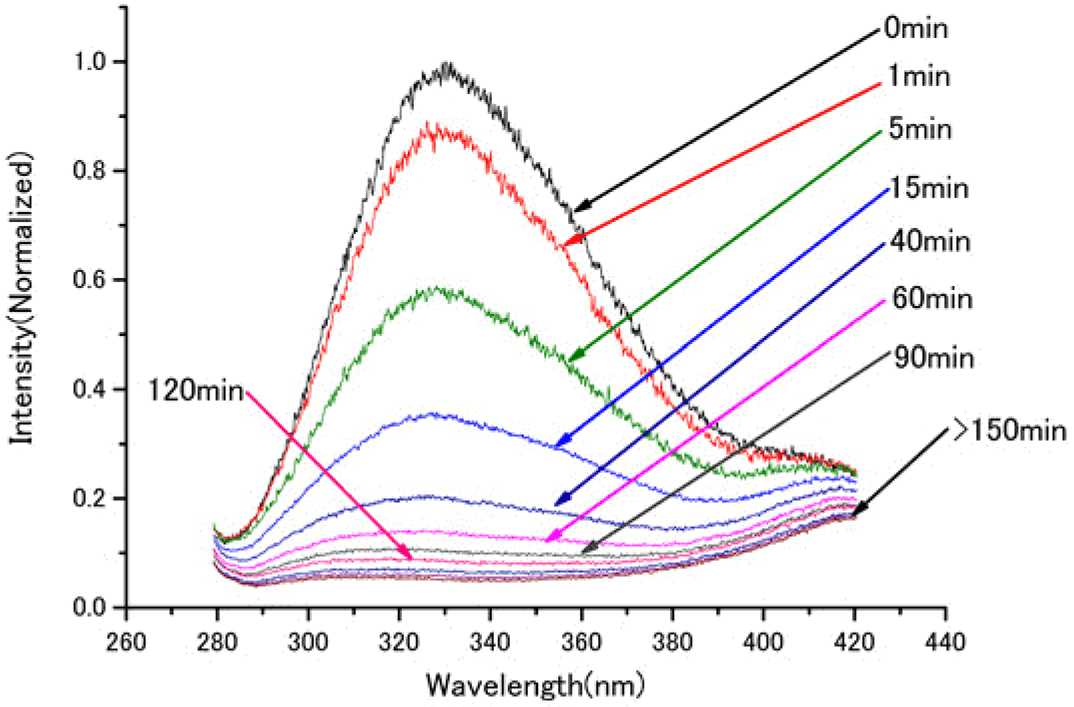

**FIGURE 4.** Time variation of peak A. These spectra were measured with 10-nsec delay time.

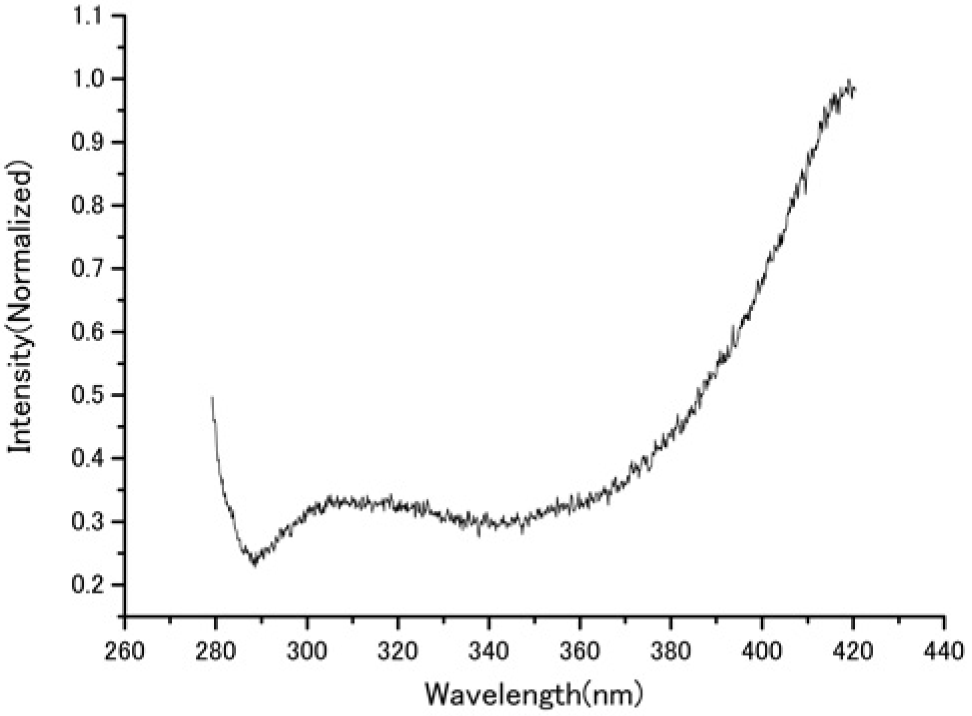

**FIGURE 5.** Fluorescence spectrum of fingerprint after 210-min illumination. This spectrum was measured with 10-nsec delay time.

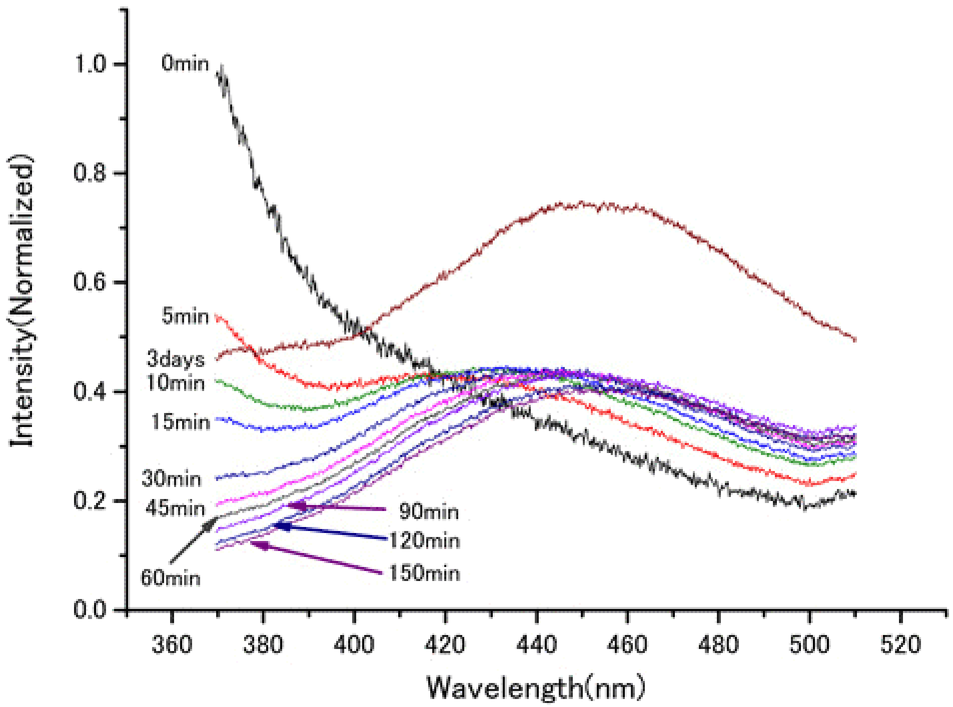

**FIGURE 6**. Time variation of peak B. These spectra were measured with 10-nsec delay time.

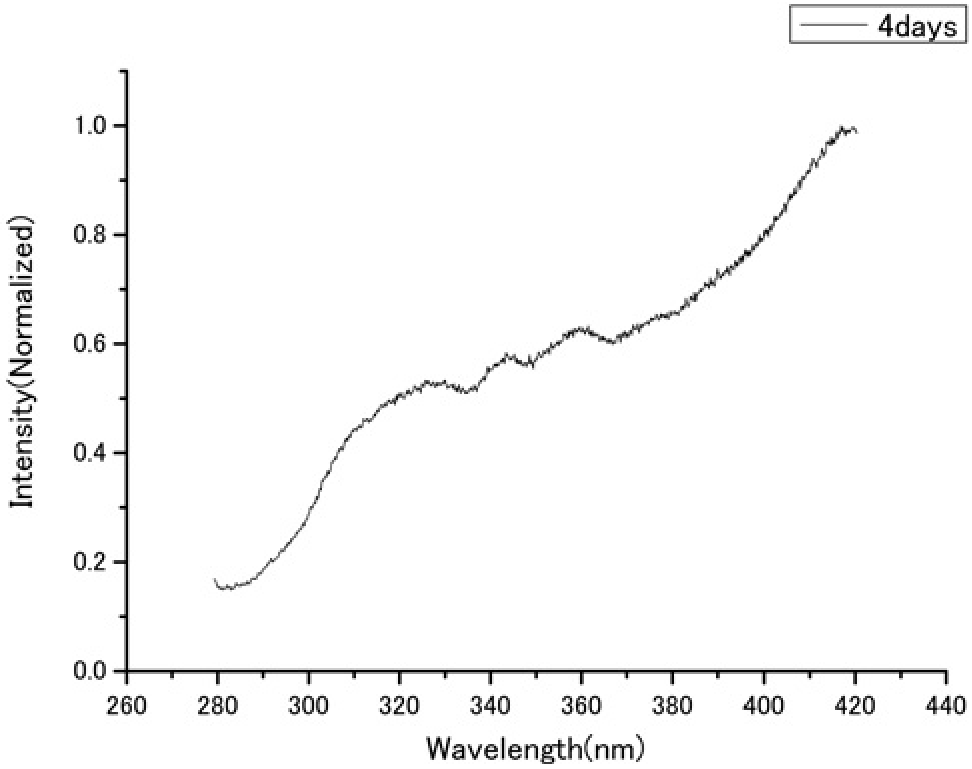

**FIGURE 7**. Spectrum of peak A after 4 days. This spectrum was measured with 10-nsec delay time.

## TIME-RESOLVED FLUORESCENCE SPECTRA OF FINGERPRINT

Next, time-resolved spectra of the fingerprints were measured to obtain the lifetime of fluorescence. Results are shown in Figs. 8 and 9 for peak A and peak B, respectively. Since the intensity of peak A decreases rapidly by 266-nm illumination, the measurement was done by pressing a new fingerprint for each measurement. For peak B, since it becomes conspicuous after enough illumination, measurements were done for the sample after 4 days. The lifetime was calculated from variation of peak area intensity with respect to the delay time. Fig. 10 shows the variation of peak area intensity, which is the total intensity from 280–420 nm for peak A and from 400–500 nm for peak B, respectively. By fitting the exponential to these intensity plots, lifetime can be estimated. Assuming that the UV fluorescence spectra consist of only one component, one exponential function was fitted to the curve. The lifetime was estimated as 2.0 nsec for peak A and 6.2 nsec for peak B, respectively. However, these values are regarded as the averaged lifetime since several components seem to exist in the spectra. In Fig. 11, the intensity profiles are shown without 10- and 15-nsec delay time. As can be seen from these figures, the peak position moves from 330 to 360 nm for peak A and from 440 to 460 nm for peak B as delay time increases. This means that there are several components and, for example, the lifetime of the 330-nm peak is smaller than that of the 360-nm peak.

## FLUORESCENCE SPECTRA OF ELAPSED FINGERPRINT

In order to study the aging effect of fingerprints, we measured fluorescence spectra of fingerprints left under ambient light for 14 days. We examined two ambient cases, one is a fluorescent lamp and the other is natural daylight at the window side in a room without direct sunlight. In the latter case, most of the UV light is considered to be absorbed by window glass and only a small amount of UV light is irradiated on the sample. Results are shown in Figs. 13 and 14. These spectra were obtained with a 10-nsec delay. In the case with the fluorescent lamp, the spectrum is similar to that of a fresh fingerprint or one that has been irradiated for a short time. The profile of peak B can be recognized, but its intensity does not become conspicuous. On the other hand, in the case of natural daylight, the spectral profile varies for peak A, that is, the peak position of peak A moves to about 360 nm. This means that the 330-nm peak component reduces its intensity due to natural daylight. For peak B, the difference between a fluorescent lamp and natural daylight is present, but small. That is, the sample left in the natural daylight shows slightly more fluorescence than that under a fluorescent lamp. This increase might be attributed to UV components in daylight through the window.

## SUMMARY

There are two main peaks, peak A and peak B, in the UV fluorescence spectra of fingerprints. The peak position of peak A is at about 330 nm at first or just after being pressed. Several components seem to exist in peak A. By measuring fluorescence spectra with more than 20-nsec delay time, these components can be revealed since the main component at the 330-nm wavelength becomes weak. Peak B is located at around 440 nm, but it appears after enough 266-nm illumination and its intensity slightly increases as time goes by. On the other hand, peak A reduces its intensity with 266-nm illumination and after about 90 min, it is almost eliminated. Therefore, peak A is dominant from just pressed to about 20 min and after 60 min, peak B becomes dominant. After 3-h illumination, peak A seems to submerge under peak B.

By leaving the fingerprint in a room under illumination of a fluorescent lamp without laser illumination, peak A can be restored partly, while the intensity of peak B still increases. Peak position also varies by 266-nm illumination, that is, from 330 to 305 nm for peak A and from 440 to 460 nm for peak B.

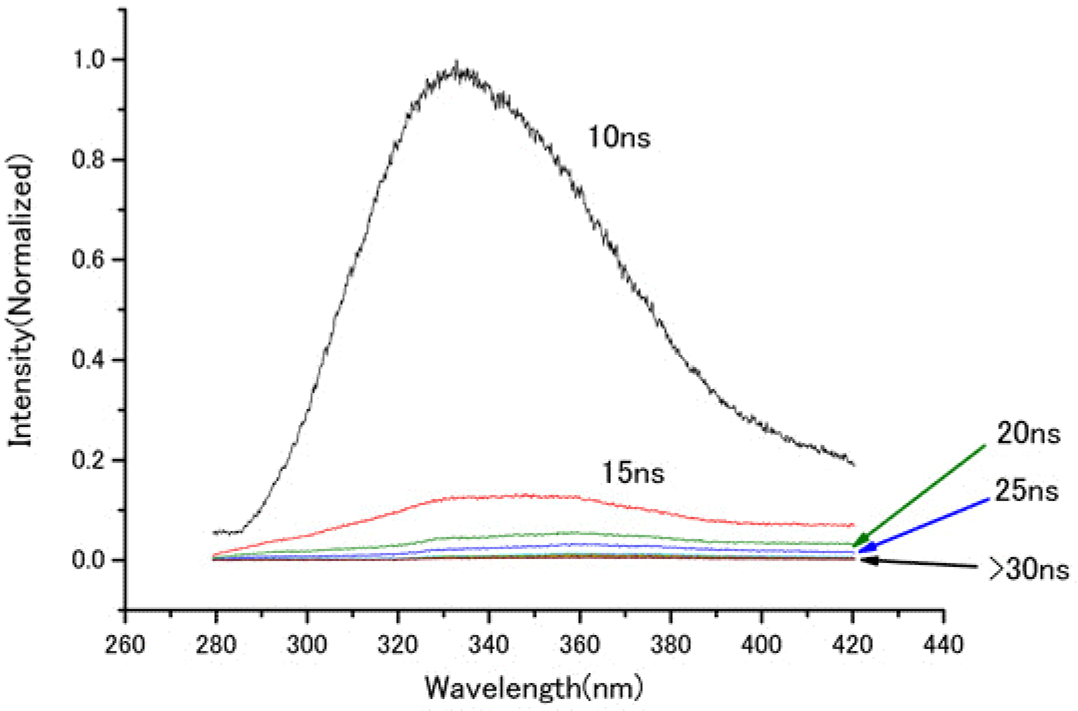

**FIGURE 8.** Time-resolved spectra of peak A.

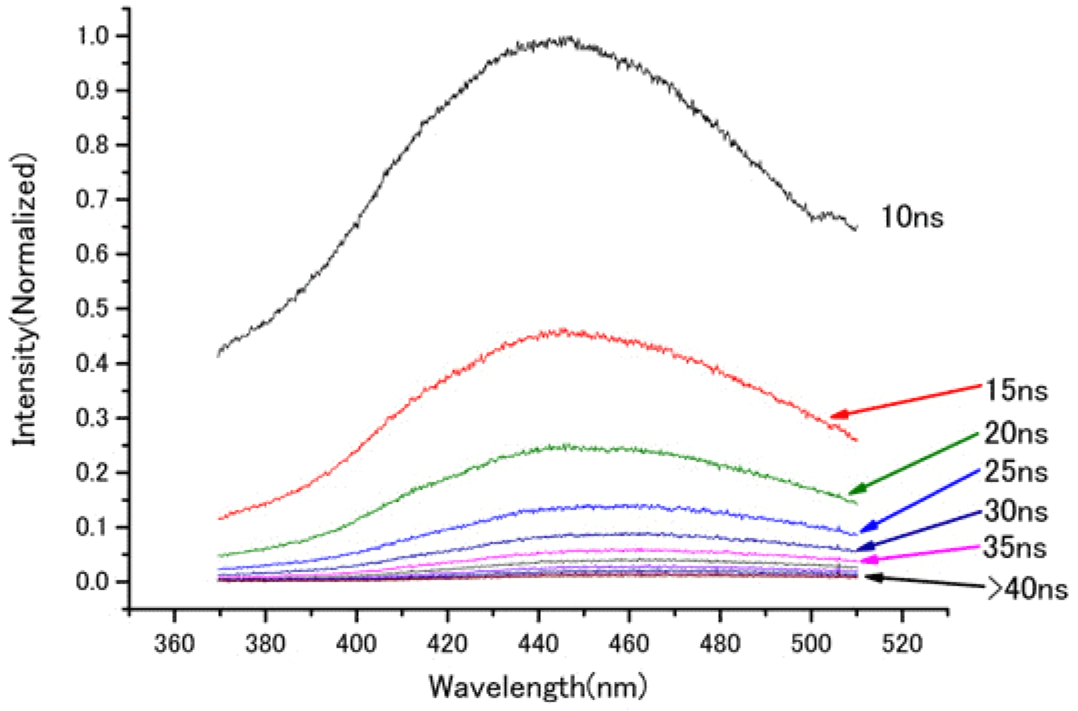

**FIGURE 9.** Time-resolved spectra of peak B.

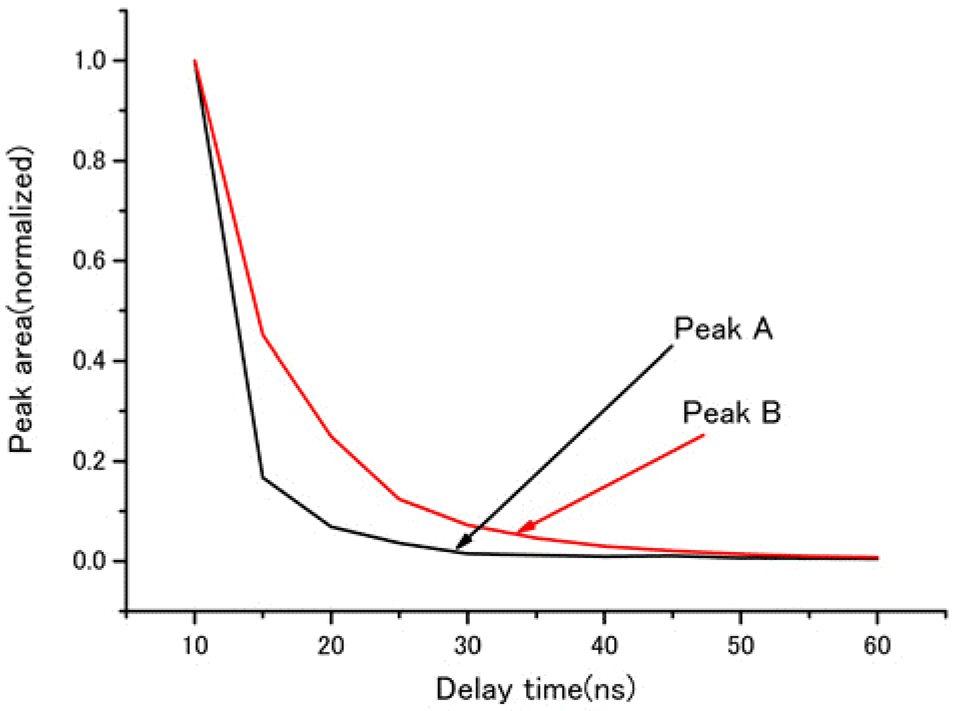

**FIGURE 10**. Fluorescence intensity of peak A and peak B with respect to the delay time.

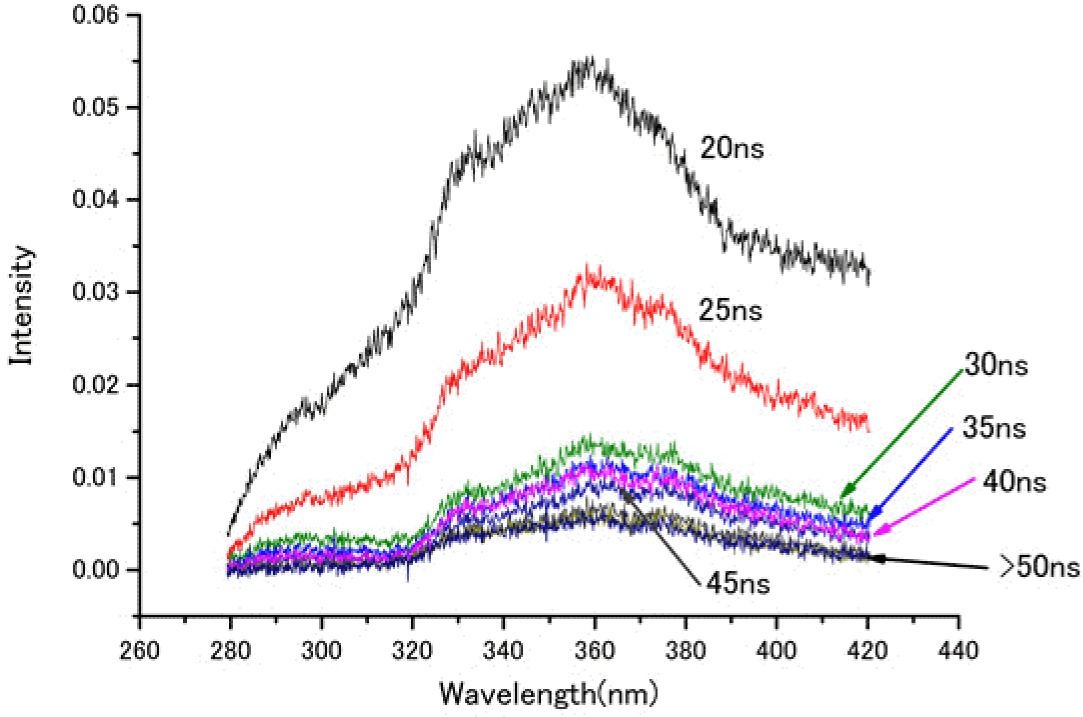

**FIGURE 11.** Time-resolved spectra of peak A. These are the same as in Fig. 8, but without spectra for 10- and 15-nsec delay time. These are shown only to inspect details of spectra with more than 20-nsec delay time.

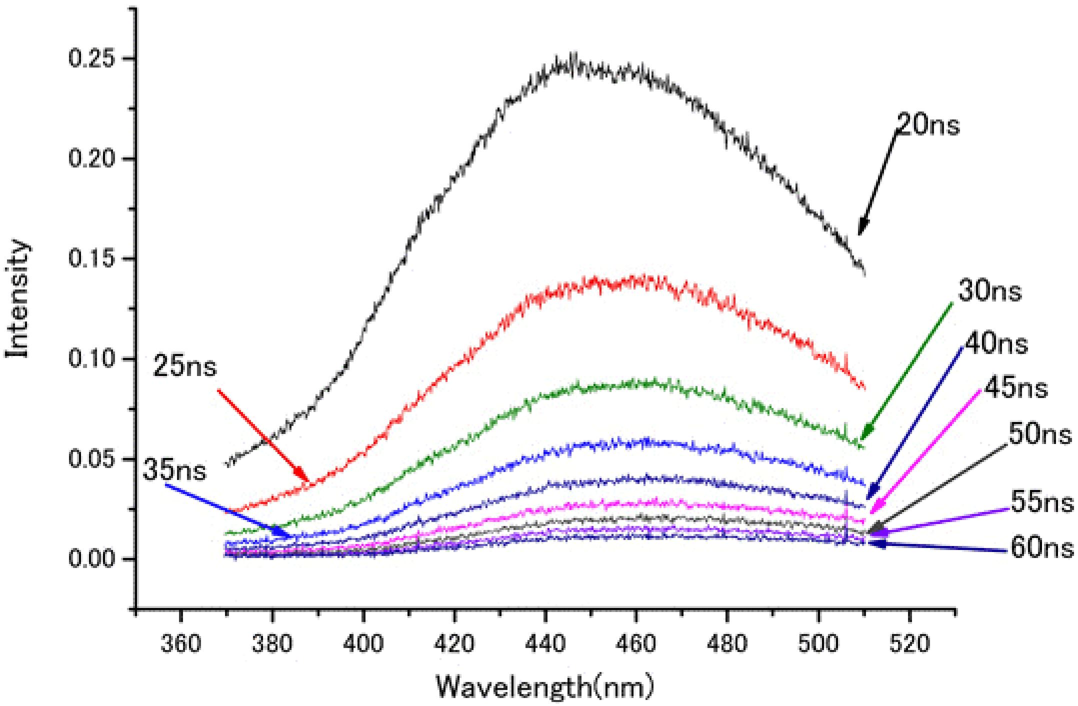

**FIGURE 12.** Time-resolved spectra of peak B. These are the same as in Fig. 9, but without spectra for 10- and 15-nsec delay time. These are shown only to inspect details of spectra with more than 20-nsec delay time.

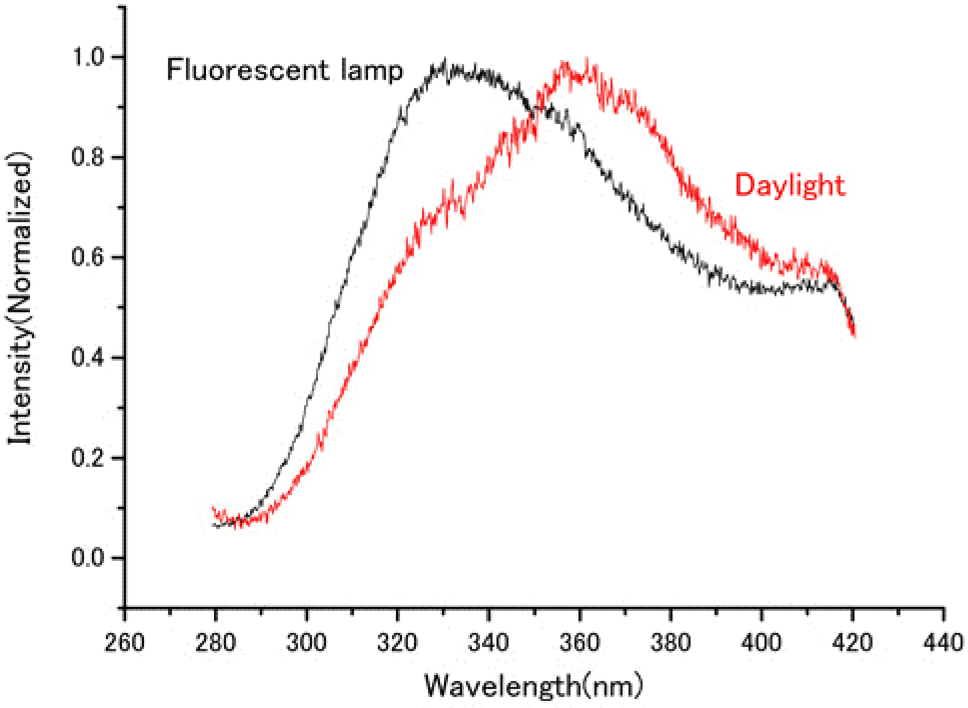

**FIGURE 13.** Fluorescence spectra of peak A after being left 14 days in ambient light.

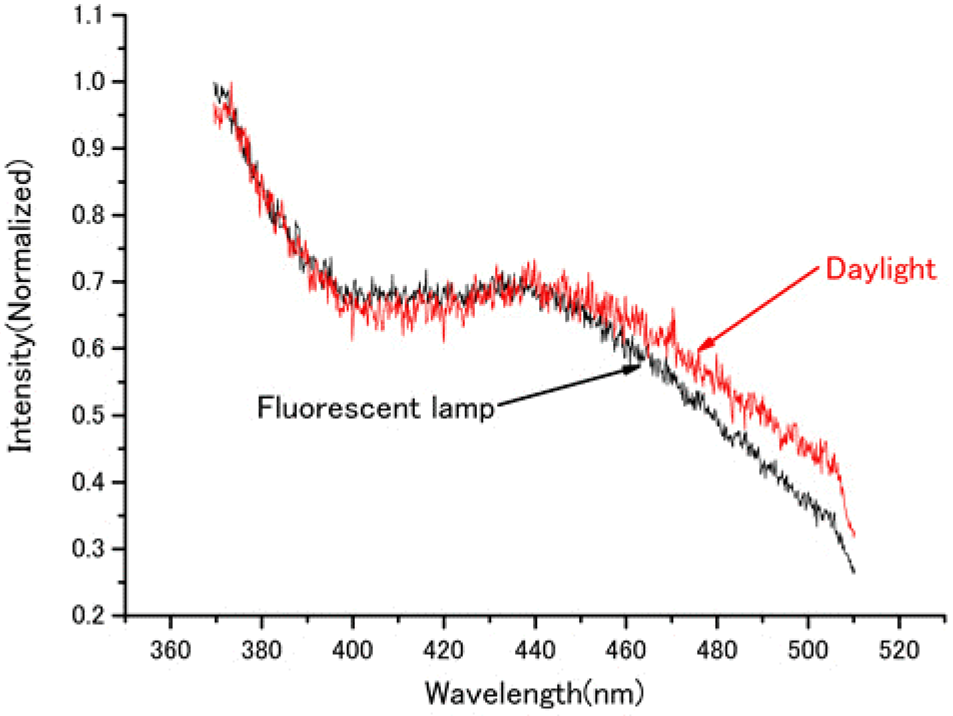

**FIGURE 14**. Fluorescence spectra of peak B after being left 14 days in ambient light.

Time-resolved fluorescence spectra were also measured for these two peaks. The lifetime of each peak is 2.0 nsec (peak A) and 6.2 nsec (peak B) on average. Both peaks consist of several components with different lifetimes. In the case of peak A, the 330-nm peak decays fast and a new component at the 360-nm wavelength becomes dominant when the delay time exceeds 20 nsec. In the case of peak B, unlike peak A, no clear peak separation is observed, but the peak position seems to move from 440 to 460 nm.

For imaging fingerprints using UV fluorescence, using peak A is appropriate because this is the dominant component within about 20 min. Imaging the peak B component might be applicable when no background fluorescence exists over the 400-nm wavelength region. Details of imaging UV fluorescence of fingerprints will be reported in a future study.

## ACKNOWLEDGMENTS

The authors would like to thank Dr. Shigeki Takeuchi of the Criminal Investigative Laboratory of Gifu Prefectural Police Headquarters for his helpful discussions and Dr. Kenro Kuroki of the National Research Institute of Police Science and Mr. Takashi Ikegami of the Forensic Science Laboratory of Shizuoka Prefectural Police Headquarters for their assistance in the experiment.

## References

1. Dalrymple, B.E., Duff, J.M., and Menzel, E.R. (1977) Inherent fingerprint luminescence-detection by laser. *J. Forensic Sci.* **22**, 106–115.
2. Menzel, E.R. (1999) *Fingerprint Detection with Lasers*. 2nd ed. [revised and expanded]. Marcel Dekker, New York.

3. Lee, H.C. and Gaensslen, R.E. (2001) Methods of latent fingerprint development. In *Advances in Fingerprint Technology*. 2nd ed. Lee, H.C. and Gaensslen, R.E., Eds. CRC Press, Boca Raton, FL.
4. Menzel, E.R. (2001) Fingerprint detection with photoluminescent nanoparticles. In *Advances in Fingerprint Technology*. 2nd ed. Lee, H.C. and Gaensslen, R.E., Eds. CRC Press, Boca Raton, FL.
5. Bramble, S.K., Creer, K.E., Gui Qiang, W., and Sheard, B. (1993) Ultraviolet luminescence from latent fingerprints. *Forensic Sci. Int.* **59**, 3–14.
6. Bramble, S.K. (1996) Fluorescence spectroscopy as an aid to imaging latent fingermarks in the ultraviolet. *J. Forensic Sci.* **41**, 1038–1041.
